# Spread of Scarlet Fever

**Published:** 1909-12-04

**Authors:** 


### Medico-Legal Points.

### SPREAD OF SCARLET FEVER.

At Willesden Petty Sessions on October 2 Mrs. Rose Hibbard was summoned at the instance of Dr. William Butler, medical officer of health of Willesden, "that she, being in charge of Stanley Hibbard whilst suffering from an infectious disease, namely, scarlet fever, wilfully exposed such sufferer without proper precautions against spreading the disease." Proceedings were taken under the Public Health Act 1875, s. 126 (2). As a result of defendant's recklessness, two other children contracted scarlet fever and were removed to the Isolation Hospital, and either of these cases might quite conceivably have terminated fatally. In similar cases legal proceedings have been taken in the High Court by parents of children who have contracted scarlet fever, and heavy damages have been awarded. On September 6 Stanley Hibbard, a son of defendant, was excluded from Furness Road School on suspicion of having chicken-pox. On the following day Miss Thompson, lady health visitor, advised Mrs. Hibbard to consult a doctor, as Stanley's symptoms were those of scarlet fever. This Mrs. Hibbard refused to do. On the 13th Miss Gaul, another lady health visitor, examined the boy and found slight desquamation of both ears. The boy was medically examined on September 16 at the school by Dr. Gore Reynolds, assistant school medical officer, who diagnosed scarlet fever, informed the boy's mother of the fact, and told her it was infectious. Later on the same day the boy was allowed to play in the street where he lived, and to go for a ride in a baker's cart with other boys. Defendant then, apparently, became alarmed and consulted her own doctor, who also diagnosed scarlet fever and notified Dr. Butler. In spite of the decided warning of the health visitors, the child was sent on the previous Sunday to the Socialist Sunday School. The Chairman said that the defendant and her husband could not be accused of ignorance, and the Bench were loth to accuse them of deliberate revolt against a law passed in the best interests of the public. But their action (and the husband was at least equally responsible) had entailed serious loss to the ratepayers and an undoubted risk of human lives. As a warning to others, the defendant would be fined 20s. and court costs. As a result of the wilful and deliberate exposure of the case after definite and precise warning as to its character several persons contracted scarlet fever, and the local authority were put to some scores of pounds of expense in isolating the patients and dealing with the outbreak. The costs of the prosecution alone borne by the Willesden Council, exceeded the penalty imposed by the magistrate, and so far from being a warning, the action of the bench amounts almost to an incitement to recklessness.

In Best *v.* Stapp (*Times*, November 9, 1872) an action was brought by a lodging-house keeper at Eastbourne, into whose house the defendants had introduced their children while infected with scarlet fever, whereby the plaintiff lost four of his own children, and incurred expense caused to him by the illness and burial of the children, the jury found that the defendants knew that their children were in an infectious state when they introduced them into the plaintiff's house, and gave the plaintiff a verdict for £120. In Managers of Metropolitan Asylum District *v.* Hill (6 A. C. 205), Lord Blackburn said: "Where those who have the custody of the person sick of an infectious disorder have not the means of isolating him from the other inmates, which is very commonly the case with the poor, and consequently those other inmates and the neighbours are exposed to the risk of infection, I think that the inability to isolate him would form a sufficient excuse to be a defence to any indictment; and I think also, though I am not aware of any authority on the subject, that the neighbours could not maintain any action for the damage which they would in such a case sustain from the proximity of the infected person, it being a necessary incident to the use of property for habitations in towns that contagious sickness may befall their neighbours. If those who have the charge of the infected person have the means of isolating him on the spot, they certainly do well to use them; and if it cannot be done on the spot, and they can, either by their own means or by the aid of charitable persons who have erected a hospital, find a place where he can be isolated so as to avoid the risk of infection, they will do well to use these means. I do not mean to express any opinion as to whether, at common law, they would or would not be responsible for not doing so; but there is no authority and, I think, no principle for saying that they are justified in removing him to a place where the neighbours would be exposed to contagion, though it may be that those neighbours would be fewer in number than the neighbours on the spot where the infection broke out; nor for saying that if that was done, and the contagion was such as to amount to a real nuisance, those neighbours might not maintain an action and obtain an injunction to protect themselves against the importation of foreign infection."

In The Tunbridge Wells Local Board *v.* Bisshopp (2 C.P.D. 187), a medical man in practice in Tonbridge sent a patient who was suffering from scarlet fever to the fever hospital there with a certificate, directing him to walk in the middle of the road, and not to talk to anyone; but in consequence of an alleged informality in the certificate the patient was refused admission, whereupon the medical man walked with him through the streets of the town to the residence of the chairman of the local board, from whom, after some delay, he obtained an order for the man's admission to the hospital. He then returned with the patient to the police-station to procure the ambulance to convey him thither. Upon an information against the medical man for an alleged infringement of the statute, the justices were of opinion that it was not proved before them that the medical man "had charge of" the patient, that he had not wilfully exposed the patient in any street or public place "without proper precaution," and that he had made the best use of the means at his disposal to prevent the spread of the fever; and they refused to convict him. It was held that their decision was right.

